# *Brucella abortus* triggers the differential expression of immunomodulatory lncRNAs in infected murine macrophages

**DOI:** 10.3389/fimmu.2024.1352306

**Published:** 2024-02-16

**Authors:** Manuel Flores-Concha, Leonardo A. Gómez, Rodrigo Soto-Shara, Raúl E. Molina, Roberto F. Coloma-Rivero, David A. Montero, Ítalo Ferrari, Ángel Oñate

**Affiliations:** Laboratory of Molecular Immunology, Department of Microbiology, Universidad de Concepción, Concepción, Chile

**Keywords:** brucella abortus, macrophages infection, gene expression, lncRNA, immunomodulation

## Abstract

**Introduction:**

The lncRNAs (long non-coding RNAs) are the most diverse group of non-coding RNAs and are involved in most biological processes including the immune response. While some of them have been recognized for their influence on the regulation of inflammatory activity, little is known in the context of infection by *Brucella abortus*, a pathogen that presents significant challenges due to its ability to manipulate and evade the host immune system. This study focuses on characterize the expression profile of LincRNA-cox2, Lethe, lincRNA-EPS, Malat1 and Gas5 during infection of macrophages by *B. abortus*.

**Methods:**

Using public raw RNA-seq datasets we constructed for a lncRNA expression profile in macrophages *Brucella*-infected. In addition, from public RNA-seq raw datasets of RAW264.7 cells infected with *B. abortus* we constructed a transcriptomic profile of lncRNAs in order to know the expression of the five immunomodulating lncRNAs studied here at 8 and 24 h post-infection. Finally, we performed *in vitro* infection assays in RAW264.7 cells and peritoneal macrophages to detect by qPCR changes in the expression of these lncRNAs at first 12 hours post infection, a key stage in the infection cycle where *Brucella* modulates the immune response to survive.

**Results:**

Our results demonstrate that infection of macrophages with *Brucella abortus*, induces significant changes in the expression of LincRNA-Cox2, Lethe, LincRNA-EPS, Gas5, and Malat1.

**Discussion:**

The change in the expression profile of these immunomodulatory lncRNAs in response to infection, suggest a potential involvement in the immune evasion strategy employed by *Brucella* to facilitate its intracellular survival.

## Introduction

Brucellosis is a worldwide spread zoonotic disease caused by Gram-negative bacteria of the genus *Brucella*, which are facultative intracellular pathogens (1, 2). There are 12 species in this genus known to infect specific mammalian hosts, and *Brucella abortus* is one of the most relevant pathogens due to its zoonotic potential and impact on livestock (3). During cell infection of the host, *B. abortus* evades its degradation in the phagolysosomes and modulate the immune response of phagocytic cells such as macrophages to develop a replicative niche within vesicles associated with the endoplasmic reticulum (ER) (4). Several virulence factors of *B. abortus* participate in this process, including their type IV secretion system (T4SS) and atypical lipopolysaccharide (LPS). Some structural differences between the *Brucella* LPS (Br-LPS) and enterobacterial LPS (e.g., *Salmonella* or *Escherichia coli*) such as the long length of the fatty acid lipid A of Br-LPS (with 19 and 28 carbons attached to a 2,3-diamino-2,3-dideoxyglucose backbone) or its net charge are related to the weak recognition by the MD2/TLR4 complex, one of the main receptors of innate immunity in the recognition of bacterial LPS, resulting in an attenuated production of proinflammatory cytokines (5–9). However, *B. abortus*-infected macrophages secrete pro-inflammatory cytokines such as IL-6 and TNF-α in human monocytes (THP-1) through a TLR-4-independent pathway, which could be mediated mainly by the TLR2 in response to surface lipoproteins (10). The secretion of IL-6 by *Brucella*-infected macrophages via TLR-2 contributes to activating the innate immune response against the infection; nevertheless, it could reduce the expression of class II MHC molecules (MHC-II) through a negative regulation of the transcriptional modulator CIITA (11). Interestingly, *B. abortus* RB51 (RB51), an attenuated rough strain lacking the O chain of LPS (12), induces the secretion of high levels of IL-6 and TNF-α and increases the expression of MHC-II (13). These findings demonstrate that various elements of *B. abortus* can finely modulate the inflammatory response and, consequently, the host’s immune response activation during this infection.

Several factors can strongly influence the inflammatory response under pathological and steady-state conditions, such as the expression of non-coding RNAs (ncRNAs), which actively participate in the modulation and regulation of the gene expression (14). Recently, Mattick et al. published a statement indicating that ncRNAs can be classified in three categories according to their size (1): Small non-coding RNAs (sncRNAs), which group transcripts of less than 50 nt (2); Medium-length transcripts, between 50-500 nt, which includes RNA polymerase II (Pol II) transcripts and RNA polymerase III (Pol III) transcripts; and (3) Long non-coding RNAs (lncRNAs), a group of RNAs generated mainly by RNA polymerase II (Pol II), with heterogeneous functions and lengths exceeding 500 nt (15). These lncRNAs can interact with DNA, RNA, and/or proteins to regulate gene expression at the transcriptional, post-transcriptional, and epigenetic levels. The great diversity of mechanisms used by lncRNAs gives them the potential to participate in the regulation of virtually all biological processes, including the immune response (16, 17). To date, approximately 20,000 lncRNA genes have been identified in humans (GENCODE version 44) and more than 14,000 lncRNA genes in mice (GENCODE version M33), the majority with unknown functions.

Some lncRNAs expressed in immune cells have been associated with the regulation of the inflammatory process (18). Examples are lincRNA-Cox2 and Lethe, which can modulate the activation of relevant transcription factors in immunity, such as NF-κB (19, 20). Moreover, lincRNA-EPS and lincRNA-Cox2 can exert epigenetic control, interacting with heterogeneous nuclear ribonucleoprotein (hnRNP) complexes and chromatin remodeling and histone deacetylase complexes to modulate genome accessibility and control the expression of hundreds of genes related to the immune response (21–23). This inflammatory environment affects the physiology of macrophages, generating a spectrum of pro-inflammatory (M1) or anti-inflammatory (M2) phenotypes (24). Thus, the expression of many lncRNAs in macrophages varies along with the differentiation and polarization of these cells (25). The overexpression of Growth arrest-specific 5 (GAS5), a lncRNA involved in arrest of normal growth, tumor suppression and more recently related to the regulation of the immune response (26, 27), can promote the polarization of macrophages toward an M1 phenotype and inhibits the polarization of M2 phenotypes (28). On the other hand, Malat1, a lncRNA first identified in tumors of patients with non-small cell lung cancer (29), is overexpressed in LPS-activated macrophage, which, when silenced, reduces the secretion of pro-inflammatory cytokines such as IL-6 and TNF-α, thus reducing cellular apoptosis (30, 31). In addition, MALAT1 cooperates with the splicing factors PTBP1 and PSF to regulate alternative splicing, a biological process that allows the generation of multiple isoforms from a single gene, complementing the functions of the restricted set of genes associated with the immune response. (32, 33). All these studies provide important evidence about the roles that lncRNAs play in maintaining cellular homeostasis and regulating various immune processes.

Although some studies have contributed to characterizing the transcriptional profile of macrophages during *B. abortus* infection, most have focused on the analysis of coding RNAs or microRNAs (34, 35). Only a couple of studies using THP-1 cells (36) or RAW264.7 cells (37) have addressed the involvement of long non-coding RNAs (lncRNAs) during *B. abortus* infection, highlighting the need for further research to understand better their roles in the immune response and pathogenesis of this infection. Thus, using raw data sets from previously published RNA-seq, the expression of five lncRNAs – lincRNA-Cox2, Lethe, lincRNA-EPS, Malat1, and GAS5 – was examined to identify key lncRNAs involved in regulating the macrophage immune response during *B. abortus* infection. These findings were experimentally validated in the RAW264.7 cell line and peritoneal macrophages during the first 12 h post-infection by qPCR, a crucial window of time used by *B. abortus* to evade phagolysosomal degradation and modulate the immune response of the infected host’s cells.

## Materials and methods

### Differential gene expression analysis using public raw RNA-Seq data sets

We delved into public RNA-seq data sets from the National Center for Biotechnology Information (NCBI) to provide a novel analysis of lncRNA transcriptional profiles in primary macrophages and the RAW264.7 cell line infected with *B. abortus*. From these data, we evaluated the expression of five lncRNAs – lincRNA-Cox2, Lethe, lincRNA-EPS, Malat1, and GAS5 – and the expression of coding genes at 8 and 24 hours (h) post-infection [before and after establishing the replicative niche at 12 h post-infection (4)]. Briefly, using the Galaxy platform (38, 39) we analyzed raw data sets corresponding to a study focused on the identification of differentially expressed coding genes by RNA-seq from bone marrow-derived macrophages (BMM) infected with *B. abortus* strain 544 for 24 h (GEO accession: GSE100914) (Hop et., 2017). At the same time, we analyzed raw RNA-seq data obtained from RAW 264.7 infected with *B. abortus* strain 2308 for 8 and 24 h (BioProject: PRJNA694452) published by Guan et al. (37). The quality of the sequences was controlled using FastQC. Subsequently, the HISAT2 program was used to map the sequencing reads obtained from the samples to the *Mus musculus* reference genome M33 (current for GRCm39) (40). Read trimming was not performed as HISAT2 runs “soft clipping” by default (41). The mapped reads were then assembled and counted using the StringTie tool (42) where we used a reference file (Long non-coding RNA gene annotation GTF file and Comprehensive gene annotation GTF file, downloaded from GENCODE, Version M33) to guide the assembly of lncRNAs and coding genes (42). Finally, a differential gene expression (DGE) analysis was performed using the DESeq2 program (43). All the unspecified parameters were kept as default. The results of the DGE analysis at BMM were plotted on a Volcano Plot while we highlight genes or transcripts with decreased expression in blue and those with increased expression in red, employing a log-fold change (LogFC) threshold for color classification. Genes and transcripts lacking statistically significant changes in expression are represented in gray, determined by a significance threshold of 0.05.

The expression of lincRNA-Cox2, lincRNA-EPS, Lethe, Malat1, and Gas5 splicing variants in RAW264.7 cells were represented in a heat map created using GraphPad Prism version 10.0.0 for Windows (GraphPad Software, Boston, MA, USA). For this, we extracted the information generated by StringTie from the normalized counts as transcripts per million (TPM) of each transcript associated with lincRNA-Cox2, Lethe, lincRNA-EPS, Malat1 and Gas5. We then identified those transcripts present in both infected and uninfected RAW264.7 cells to calculate the log2 infected:uninfected ratio and defined it as Log2 (Fold Change TPM) (**Supplementary Data 2**). Moreover, those transcripts whose absolute value of Log2 (FC_TPM) was calculated to be greater than 2 were considered significant variations in expression and were plotted on the Venn diagram. Venn diagrams were created using BioVenn (44).

### Interaction networks

Using information available in the lncHUB2 database (45), we constructed a list of genes correlated with each of the lncRNAs studied here. From the 53453 genes correlated with each of the lncRNAs under study, we selected a maximum of 1000 genes for each lncRNA, prioritizing those with the highest Pearson correlation coefficient and restricting the selection only to genes with coefficients greater than 0.1 to discard those whose correlation is negligible (46). Subsequently, from the list of coding genes differentially expressed in the transcriptome of RAW264.7 infected with *B. abortus* at 8h post infection, obtained as described in the previous section, we selected those correlated with the lncRNAs under study, based on the list created with the correlation data obtained from lncHUB2. Then, the list of common genes was used as input in the Network Analysis tool of the InnateDB server (https://www.innatedb.com/) (47), with the aim of generating a network of interactions between genes correlated with the lncRNAs studied and immunity-related genes present in the curated InnateDB data list.

### Animal studies

Female BALB/c mice aged six to eight weeks were obtained from the Institute of Public Health (ISP, Santiago, Chile). The animals were kept under controlled temperature conditions and with *ad libitum* access to food and water. All experimental procedures and animal care were performed following the guidelines of the institutional bioethics and biosafety committee (Certificate CEBB 1466-2023).

### Bacterial strains and culture conditions

The *B. abortus* 2308 (S2308) and *B. abortus* RB51 (RB51) strains were obtained from the culture collection of the Laboratory of Molecular Immunology, Department of Microbiology, University of Concepcion. For infection assays, S2308 and RB51 were cultured in *Brucella* broth and incubated with agitation (120 RPM) at 37°C for 48 h. The RB51 culture was supplemented with Rifampicin as a selection agent. Heat-killed *B. abortus* 2308 (HKBA) was obtained by incubating a suspension of S2308 at 70°C for 30 minutes (24).

### Cell line and primary macrophage cell culture

The RAW 264.7 macrophage cell line (ATCC^®^ TIB-71™) was cultured in DMEM medium (Gibco BRL, USA) supplemented with 10% fetal bovine serum (FBS) (Gibco BRL, USA), 100 µL/mL of penicillin, and 100 µg/mL of streptomycin (Invitrogen™, Auckland, New Zealand). The cells were incubated at 37°C with a 5% CO_2_ atmosphere. Primary cultures of murine macrophages were obtained from the peritoneal cavity (48). The cells were adjusted to 1x10^6^ peritoneal cells per mL and then seeded in 12-well plates (1 mL per well) and incubated at 37°C with 5% CO_2_ for 24 h in RPMI-1640 medium supplemented with 10% FBS and an antibiotic-antimycotic solution (Sigma-Aldrich). After incubation, the non-adherent cells in the culture flask were removed by washing three times with PBS. Finally, the adherent cell monolayer was incubated without antibiotic-antimycotic under the previously mentioned conditions for infection assays.

### Infection assay

RAW 264.7 or peritoneal macrophages were collected and seeded in 12-well plates (1 mL per well) at a concentration of 1x10^6^ cells per mL in DMEM or RPMI medium, respectively. The plates were incubated for 2 h at 37°C and 5% CO_2_ to ensure the adherence of these cells to the surface of the plate. Once adhered, the cell culture medium was replaced with S2308, RB51, or HKBA suspended in DMEM at a multiplicity of infection (MOI) of 100:1 (bacteria:macrophages) or in RPMI at an MOI of 10:1, respectively. Subsequently, the plates were centrifuged for 5 minutes at 350 x g at room temperature to synchronize the infection. Afterwards, the cultures were incubated for 1 h at 37°C and 5% CO_2_. The adherent cells were washed twice with PBS and treated for 1 h in DMEM or RPMI with 50 μg/mL of gentamicin to eliminate extracellular bacteria. After the incubation, the gentamicin was removed and replaced with fresh medium (36). The cells were incubated for 1, 6, and 12 h post-infection at 37°C and 5% CO_2_ to perform transcriptional expression studies and evaluate cytokine levels. In each experiment, unstimulated macrophages were used as the negative control, and macrophages stimulated with 0.1 µg/mL of LPS (Sigma-Aldrich) were used as the positive control of inflammatory response induction. All conditions were performed in triplicate.

### LncRNA expression

The differential expression of long noncoding RNAs (lncRNAs) was evaluated using total RNA in RAW 264.7 cells infected for 1, 6, and 12 h and in peritoneal macrophages at 12 h post-infection. The total RNA was extracted using TRIzol according to the manufacturer’s protocol. 5 µg of RNA was converted to cDNA for each condition using the Maxima First Strand cDNA Synthesis Kit for RT-qPCR with dsDNase following the manufacturer’s instructions. The transcriptional expression of the lncRNAs lincRNA-Cox2, Lethe, lincRNA-EPS, Gas5, and Malat1 in infected or stimulated macrophages was determined by RT-qPCR using 100 ng of cDNA from each sample per well (49). The relative expression of these transcripts was determined based on their expression in untreated cultures, using the expression of GapdH as a reference gene. Per the manufacturer’s instructions, the PCR reaction was performed using the Takyon™ qPCR for SYBR^®^ kit (Eurogentec, Seraing, Belgium). The sequence of the primers used in this study is detailed in **Table 1**. The expression levels evaluated by qPCR were calculated based on the average of the data obtained from the analysis of two technical replicates of each sample.

**Table 1:** Primers used for this study.

LncRNA	Associated function	Forward	Reverse	Reference
LincRNA-Cox2^1^, Ptgs2os2^2^, ENSMUSG00000097754^3^	Multifunctional regulator of inflammatory genes	TCCTTTCCCCCTCAATTCTT	TTTTCCCAATCTGCTTTGGT	(20)
Lethe^1^, Rps15a-ps4^2^, ENSMUSG00000083757^3^	Inflammation, Pre-transcriptional regulation	ACAATGAAGCCAAACTGCCG	AGTTTGTCCAAGGGACCCCA	(50)
LincRNA-EPS^1^, Ttc39aos1^2^, ENSMUSG00000085873^3^	Inflammation, epigenetic regulation	GCGCACTTCTCTCATCTGTG	TCAGCTGTAGGATGGGAGGT	(51)
GAS5 (Mus)^1,2^, ENSMUSG00000053332^3^	Macrophage polarization, Apoptosis	AGAAATGCAGGCAGACCTGT	GCACTCTAGCTTGGGTGAGG	(52)
Malat1 (Mus)^1^, NEAT2^2^, ENSMUSG00000092341^3^	Macrophage polarization, autophagy	GAGCTCGCCAGGTTTACAGT	AACTACCAGCAATTCCGCCA	(53)
GapdH (Mus)^1^, ENSMUSG00000057666^3^	Reference gene	GTGCTCTCTGCTCCTCCCTGT	CGGCCAAATCCGTTCACACCG	(54)

(^1^Name; ^2^MGI Symbol; ^3^Ensembl ID).

### Production of cytokines

The inflammatory response in RAW264.7 macrophages and murine peritoneal cavity-derived macrophages infected with *B. abortus* or their respective controls was evaluated by measuring IL-6 and TNF-α cytokine levels in the supernatant at 1, 6, and 12 h. The concentrations of these pro-inflammatory cytokines were detected using a sandwich ELISA kit following the manufacturer’s specifications (Invitrogen). The absorbance was read on a Tecan nano-pro ELISA reader at 490 nm. The cytokine concentrations (pg/ml) were obtained by developing standard curves using recombinant IL-6 and TNF-α. All experiments were performed in triplicate.

### Statistical analysis

All graphs and statistical analyses were performed using GraphPad Prism version 10.0.0 for Windows (GraphPad Software, Boston, MA, USA). A one-way or two-way ANOVA determined significant differences between groups. Multiple comparisons were made using the Bonferroni test. P values under 0.05 were considered statistically significant.

## Results

### Differential expression analysis of lncRNAs by RNA-seq reveals an upregulated expression of lincRNA-Cox2 in *B. abortus*-infected BMM

**Figure 1:**
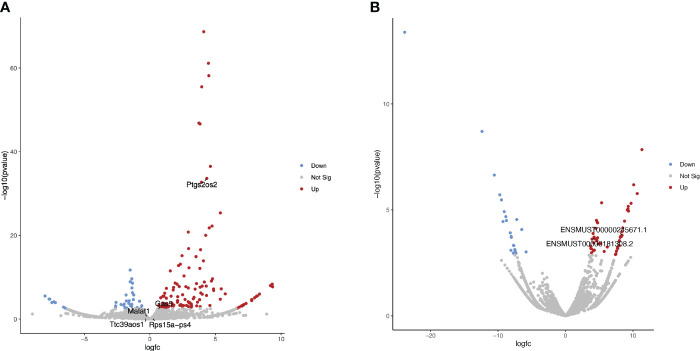
Differential expression analysis of lncRNAs in *B abortus*-infected BMM at 24 h The volcano plots present the results of a differential expression analysis for **(A)** lncRNA genes and **(B)** lncRNA transcripts. These were identified by performing bioinformatic analysis on publicly available raw sequencing data (GEO accession: GSE100914) derived from total RNA samples extracted from bone marrow macrophages (BMM) infected with *Brucella abortus* strain 544. The plots highlight downregulated genes or transcripts in blue and upregulated ones in red, using a log-fold change (LogFC) threshold for color coding. Genes and transcripts that do not exhibit a statistically significant change in expression are depicted in gray based on a significance threshold of 0.05.

An initial exploration of lncRNA expression induced by *Brucella* infection of a primary line of murine macrophages was performed. Public raw RNA-seq data sets (GEO accession: GSE100914) were processed and analyzed to determine the transcriptional landscape of lncRNAs, including Lethe, lincRNA-Cox2, lincRNA-EPS, Malat1, and GAS5 (**Supplementary Data 1**). The results show that 36 lncRNAs were significantly (p<0.05) downregulated in these cells, while 129 lncRNAs were significantly (p<0.05) upregulated during *Brucella* infection. Although the abundance of some lncRNAs with immunomodulatory activity, such as lincRNA-EPS, Lethe, Malat1, and Gas5, does not change significantly at 24 h post-infection, lincRNA-Cox2 (*Ptgs2os2*) was highly overexpressed in these cells during the *Brucella* infection (**Figure 1A**). Moreover, we identify two lincRNA-Cox2 splicing variants (ENSMUST00000245671.1 and ENSMUST00000181308.2), which were significantly increased in abundance in *B. abortus*-infected BMM (**Figure 1B**).

### Analysis of transcriptomic abundance for lincRNA-Cox2, lincRNA-EPS, Lethe, Malat1, and Gas5 in *B. abortus*-infected RAW264.7 macrophages reveals differences at 8 and 24 h post-infection

**Figure 2:**
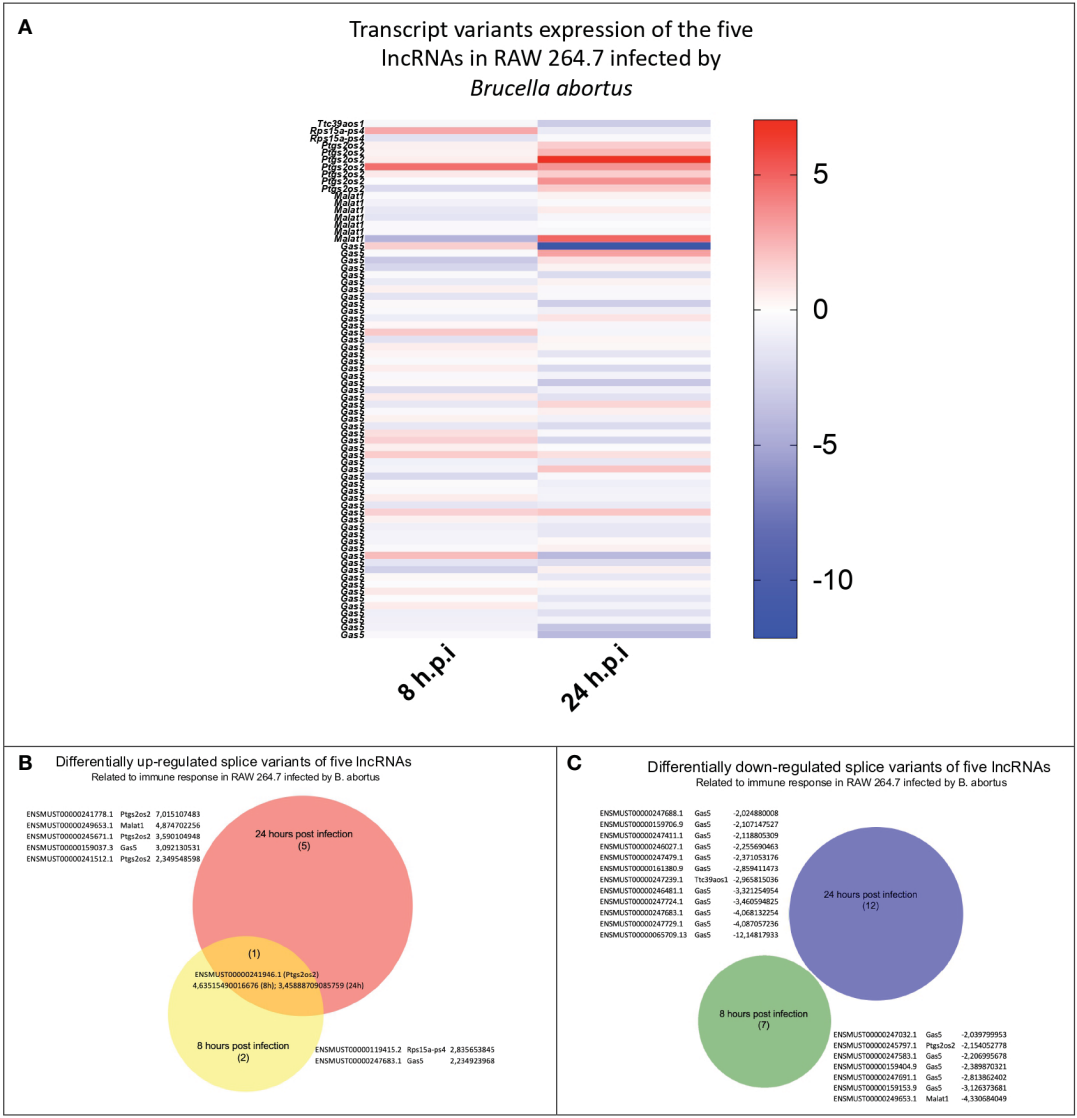
Identification and comparison of the expression of splicing variants of the five evaluated lncRNAs during *B abortus*-infected RAW264.7 macrophages. The expression of 72 splicing variants of five lncRNAs related to the immune response detected in *B abortus*-infected RAW264.7 macrophages were analyzed at 8 and 24 h post-infection. **(A)** Heat Map shows the log2 (fold change) calculated by TPM of each transcript, where blue corresponds to values of downregulated transcripts, and red corresponds to upregulated transcripts, both compared to the uninfected control. The Venn diagram shows the transcript_id, gene_symbol, and log2 (fold change TPM) of splicing variants that are upregulated **(B)** or downregulated **(C)**.

Knowing the global expression of lncRNA in *B. abortus*-infected BMM at 24 h, we proceeded to study the specific expression of five lncRNA: lincRNA-Cox2, lincRNA-EPS, Lethe, Malat1, and Gas5 in the RAW264.7 cell line at 8 and 24 h post-infection. Public raw RNA-seq data sets (https://www.ncbi.nlm.nih.gov/sra/PRJNA694452) were processed and analyzed to obtain the expression profile of these specific immunomodulatory lncRNAs (**Supplementary Data 2**). The results show that of the 72 transcripts analyzed (**Figure 2A**), the only one that maintained the trend showing an increased expression at both times evaluated was the variant ENSMUST00000241946.1 (lincRNA-Cox2 gene) (**Figure 2B**). At 8 h post-infection, the splicing variants ENSMUST00000119415.2 (Lethe), ENSMUST00000247683.1 (*Gas5*) and ENSMUST00000241946.1 (lincRNA-Cox2) were significantly overexpressed compared to the uninfected group (**Figure 2B**), while five splicing variants of the Gas5, one of Malat1 splicing (ENSMUST00000249653.1) and one of the lincRNA-Cox2 splicing (ENSMUST00000245797.1) were downregulated (**Figure 2C**). On the other hand, at 24 h post-infection, three splicing variants of the *Ptgs2os2* gene (ENSMUST00000241778.1, ENSMUST00000245671.1, and ENSMUST00000241512.1), one of the Malat1 gene (ENSMUST00000249653.1), and one of the Gas5 gene (ENSMUST00000159037.3) were overexpressed in infected RAW264.7 (**Figure 2B**). Besides, eleven splicing variants of the Gas5 gene and one of the lincRNA-EPS (ENSMUST00000247239.1) showed a lower expression than the non-infected control (**Figure 2C**). These analyses showed that during the infection by *B. abortus*, the RAW 264.7 cell population simultaneously expressed several splicing variants of each of the five lncRNAs evaluated, showing different expression profiles between 8 and 24 h post-infection.

### Interaction networks between immune-related genes and genes correlated with immunomodulatory lncRNAs

**Figure 3:**
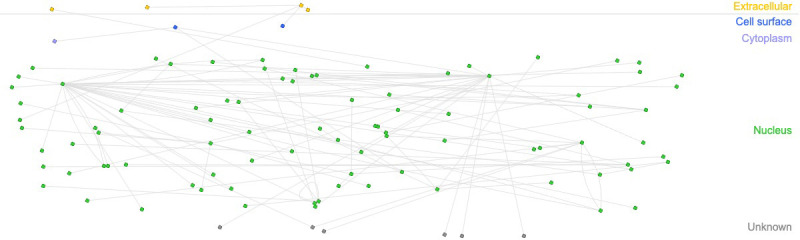
Interaction networks between immune-related genes and genes correlated with immunomodulatory lncRNAs. Interaction networks of 49 genes related to the lncRNAs analyzed in this study and overexpressed in macrophages infected with *B abortus* 2308 after 8 h post-infection. The figure shows 73 interactions between genes related to the immune response according to the InnateDB database.

Our transcriptomic analysis shows that in response to infection, there are changes in the expression profile of lincRNA-Cox2, Lethe, lincRNA-EPS, Gas5 and Malat1. In order to find potential regulatory roles of immunity-related gene activity, we analyzed the same data to, this time, identify changes in the expression of coding genes. We then used this information to construct a network of interactions between genes correlated with these lncRNAs and differentially expressed immune-related genes in RAW264.7 macrophages infected with *B. abortus* after 8 h post-infection (**Figure 3**). Of the 475 differentially expressed coding genes, we found that 49 correlate with at least one of the lncRNAs studied here. When analyzing interaction networks of these 49 genes with genes related to the immune response, we found a total of 73 interactions, suggesting that these lncRNAs could exert regulatory roles of the immune response mainly at the nuclear level (**Supplementary Data 3**).

### Analysis by qPCR detected that lincRNA-Cox2, Lethe, and lincRNA-EPS are overexpressed in *B abortus*-infected RAW.264.7 at 12 h

**Figure 4:**
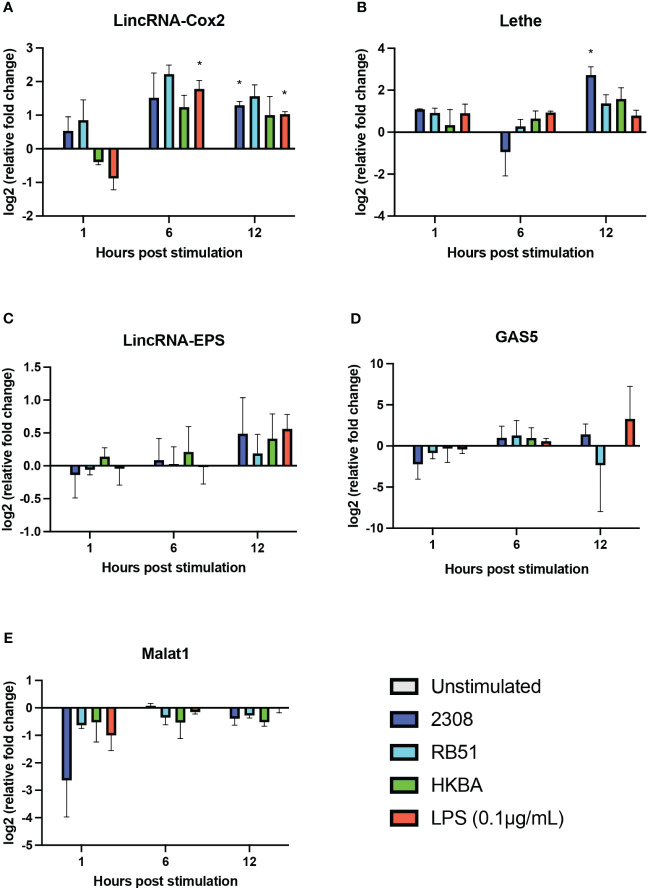
Expression of five lncRNAs related to the regulation of the inflammatory response in *B abortus*-infected RAW 264.7 macrophages. The transcriptional expression of lincRNA-Cox2 **(A)**, Lethe **(B)**, lincRNA-EPS **(C)**, GAS5 **(D)**, and Malat1 **(E)** was analyzed using RT-qPCR in RAW 264.7 macrophages infected with *B abortus* 2308 (2308), *B abortus* RB51 (RB51), or heat-killed *B abortus* 2308 (HKBA) at a multiplicity of infection (MOI) of 100:1 for 1, 6, and 12 h LPS was used as the control for the expression of the five lncRNAs. GapdH was used as the reference gene, and significant differences in expression were evaluated compared to the unstimulated control group. Asterisks indicate significant differences in expression compared to the unstimulated control group (*P < 0.05).

Considering the differences observed in the expression profile of the analyzed lncRNAs in *B. abortus*-infected RAW264.7 macrophages at 8 and 24 h post-infection, we examined the expression levels of lincRNA-Cox2, Lethe, lincRNA-EPS, GAS5, and Malat1 by RT-qPCR after 1, 6, and 12 h post-infection. The results showed that 1 and 6 h post-infection with S2308, RB51, or stimulation with HKBA did not significantly affect the expression of lincRNA-Cox2, Lethe, lincRNA-EPS, GAS5, and Malat1 in RAW264.7 macrophages compared to the unstimulated group (**Figure 4**). However, at 12 h post-infection with S2308, a significant increase in the expression of lincRNA-Cox2 (**Figure 4A**) and Lethe (**Figure 4B**) was detected. These results demonstrate that at 12 h post-infection with *B. abortus*, macrophages simultaneously overexpress lncRNAs with pro- and anti-inflammatory activity.

### Expression analysis by RT-qPCR revealed that lincRNA-Cox2 is over-expressed, and GAS5 and Malat1 are downregulated in *B. abortus* 2308-infected peritoneal macrophages at 12 h post-infection

**Figure 5:**
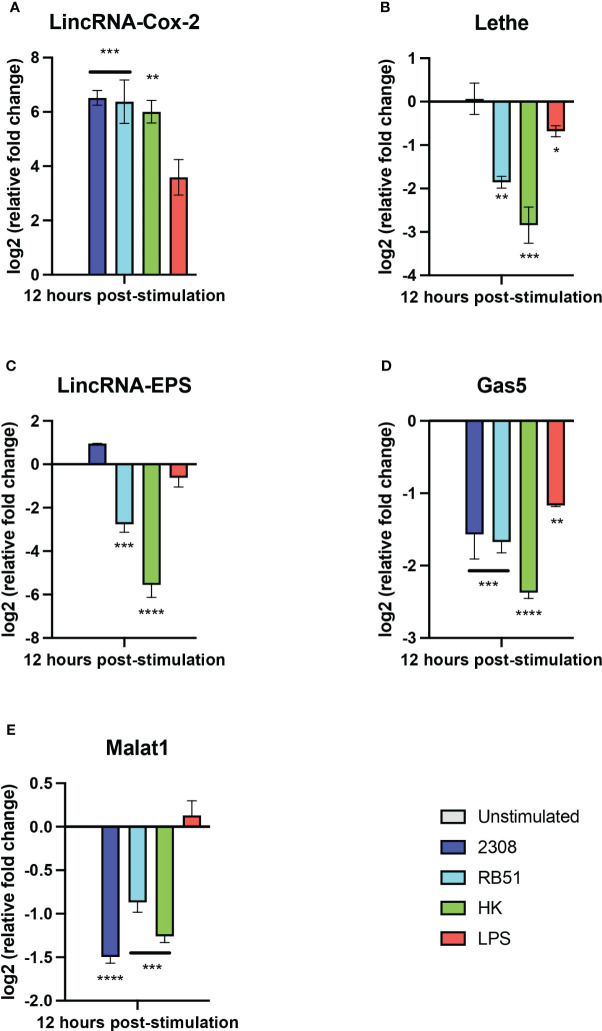
Expression of lncRNAs in peritoneal macrophages infected with *B abortus*. The expression of **(A)** lincRNA-Cox2, **(B)** lincRNA-EPS, **(C)** Lethe, **(D)** Gas5, and **(E)** Malat1 was analyzed by RT-qPCR in peritoneal macrophages treated for 12 hours with S2308, RB51, HKBA (MOI = 100) or LPS. GapdH was used as a reference gene, and significant differences in expression change were compared to the unstimulated control group (*P < 0.05; **P < 0.01; ***P < 0.001; ****P < 0.0001).

Some long non-coding RNAs (lncRNAs) have emerged as key players in the regulation of immune processes against bacterial infection. To date, no studies have described the expression of lncRNA in the primary culture of macrophages during the infection with *Brucella*. Thus, considering our results obtained in RAW264.7 macrophages, the expression of lincRNA-Cox2, Lethe, and lincRNA-EPS, Gas5, and Malat1 was studied in primary macrophages obtained from the peritoneal cavity at 12 h post-infection using RT-qPCR. The results show that the relative expression of lincRNA-Cox2 was significantly higher in all groups compared to the unstimulated group (**Figure 5A**). In addition, the expression of lincRNA-Cox2 in macrophages infected with S2308, RB51, and HKBA was even higher than in the LPS group (**Figure 5A**). The expression of Lethe was not affected in *B. abortus* 2308-infected macrophages; however, in all other groups, a downregulation was observed compared to the unstimulated group (**Figure 5B**). When evaluating the relative expression of lincRNA-EPS, it was observed that the RB51 and HKBA groups were significantly downregulated compared to the unstimulated control (**Figure 5C**). On the other hand, all experimental groups showed a negative expression of GAS5 compared to the unstimulated group (**Figure 5D**). Finally, the expression of Malat1 was significantly lower in the S2308, RB51, and HKBA groups than in the unstimulated group (**Figure 5E**). These results also show that *B. abortus* infection positively modulates the expression of lincRNA-Cox2 and downregulates the expression of GAS5 and Malat1. While the expression of Lethe and lincRNA-EPS does not change in response to S2308 infection, infection with RB51 or stimulation with HKBA significantly reduces their expression, suggesting that these lncRNAs are involved in the infection caused by *B. abortus*.

### Pro-inflammatory cytokines secreted by *B abortus*-infected RAW 264.7 cells

**Figure 6:**
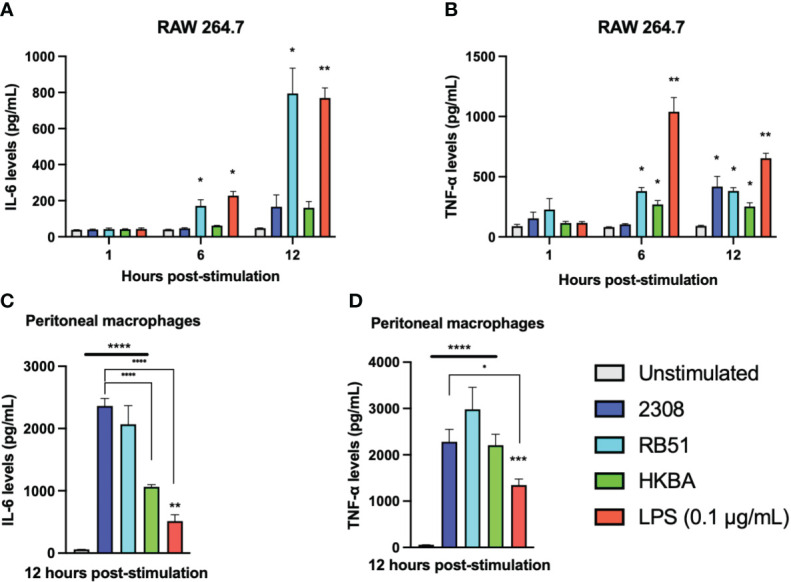
Secretion of pro-inflammatory cytokines. The levels of IL-6 **(A)** and TNF-α **(B)** secreted by RAW 264.7 macrophages infected with S2308 (2308), RB51, stimulated with HKBA, or stimulated with LPS after 1, 6, and 12 h were quantified by ELISA. Furthermore, the levels of IL-6 **(C)** and TNF-α **(D)** secreted in the culture medium of peritoneal macrophages 12 h after being treated with S2308, RB51, HKBA, or LPS were measured by ELISA. The results are expressed as the mean ± standard deviation of three replicates. The asterisks indicate significant differences compared to the unstimulated control (*P < 0.05; **P < 0.01; ***P < 0.001; ****P < 0.0001). The lines under the asterisks with vertical ends indicate differences between the two groups, and the straight lines under the asterisks indicate differences compared to the unstimulated group.

We previously detected that some lncRNAs with pro- and anti-inflammatory activity are differentially expressed in macrophages during the first 12 h of infection with *B. abortus*. Therefore, we evaluated the production of IL-6 and TNF-α as inflammation markers in RAW 264.7 cells infected for 1, 6, and 12 h with S2308, RB51, or HKBA. In addition, the production of both cytokines was also evaluated in peritoneal macrophages 12 h after exposure to the same stimuli evaluated in RAW 264.7 macrophages (**Figure 6**). The results show that after 1 h of stimulation, the IL-6 levels produced by RAW 264.7 cells did not change significantly. At 6 and 12 h post-infection, a significant increase in IL-6 was observed in RAW 264.7 infected with RB51 and in the LPS-stimulated group, whereas infection with S2308 or stimulation with HKBA did not induce significant changes in IL-6 production during the times evaluated (**Figure 6A**). Regarding TNF-α production, no significant differences were detected in any of the groups evaluated during the first hour post-stimulation. However, a significant increase was observed at 6 h of infection in the groups of cells infected with RB51, HKBA, and the LPS control. This significant increase in TNF-α level was also observed at 12 h post-stimulation, the only time evaluated at which we detected a significant increase in TNF secretion in the S2308-infected group (**Figure 6B**).

Unlike RAW cells, peritoneal macrophages significantly increased IL-6 secretion after being infected with S2308. Higher levels of IL-6 relative to the unstimulated control in peritoneal macrophages were also detected in response to RB51 infection or HKBA stimulation. In addition, the production of IL-6 was relatively similar between the groups infected with S2308 and RB51, with significantly higher levels than those stimulated with HKBA or the LPS stimulus (**Figure 6C**). On the other hand, the concentration of TNF-α induced by infection with S2308, RB51, and HKBA was significantly higher compared to the unstimulated group. They were statistically similar when comparing the production of TNF-α between the group infected with S2308, RB51, and the HKBA group. In addition, the secretion of TNF-α in peritoneal macrophages infected with S2308 was significantly higher than the group stimulated with LPS (**Figure 6D**). Based on IL-6 and TNF-α secretion, the data suggest that compared to infection with RB51, the inflammatory response induced by S2308 in RAW264.7 cells is more delayed. Furthermore, in response to infection, peritoneal macrophages appear to be better producers of proinflammatory cytokines than RAW 264.7 cells.

## Discussion

The immune response to bacterial pathogens is a complex process involving the activation and modulation of various genes and metabolic pathways (55). In particular, *B. abortus* infection presents a significant challenge for medicine and research due to the ability of this bacterium to manipulate and evade the host’s immune system (56). Long non-coding RNAs (lncRNAs), a class of non-coding RNAs, have recently emerged as key regulators of the immune response against pathogenic infections (57). In this study, by public raw RNA-seq data set analysis and qPCR detection, we have examined the expression of lncRNAs in primary mouse macrophages and RAW264.7 cell line in response to *B. abortus* infection, providing new insights into the modulation of the immune response during the early hours of *B. abortus* intracellular cycle, a critical period for the intracellular survival of the pathogen (58). Here, we focus on the expression profile of lincRNA-Cox2, Lethe, lincRNA-EPS, Malat1, and Gas5, known for their involvement in regulating gene expression related to the immune response in different inflammatory models.

Until now, only a few studies have focused on the expression of lncRNAs during *B. abortus* infection and none of them have explored the expression profile of these RNAs using primary macrophage lines (36, 37). To supplement this lack of data, we used public raw RNA-seq data sets, previously used by their authors to obtain the transcriptional profile of coding genes of bone marrow-derived macrophages (BMM) at 24h post-infection with *B. abortus* 544 (34), to identify differentially expressed lncRNAs during the infection of BMM. The analysis revealed that 165 lncRNAs were differentially expressed in BMM in response to *B. abortus* infection (**Supplementary Data 1**), similar to the 130 differentially expressed lncRNAs previously reported using RAW264.7 cells (37). Among the five lncRNAs with known immunomodulatory activity examined in this study, lincRNA-Cox2 was the only one differentially expressed 24 h post-infection in this macrophage model. Notably, its overexpression ranked it among the top 10 most differentially expressed lncRNAs following infection (**Figure 1A**). LincRNA-Cox2 can regulate various processes, such as inflammation and autophagy, by activating and repressing gene expression (20, 59). however, this is the first time that its differential expression in response to a *Brucella* infection has been reported. Alternative splicing allows for the availability of multiple isoforms of the same gene, supplementing the functions of the limited repertoire of genes related to the immune response (32). Recently, it has been reported that some pathogens, such as *Mycobacterium tuberculosis*, regulate alternative splicing in macrophages (60), and it is evident that the splicing process impacts the outcome of any infection (61). Among the 9 known lincRNA-Cox2 splicing variants, we found 2 to be differentially overexpressed in BMM infected by *B. abortus* (**Figure 1B**). Future studies could focus on determining the role of this lncRNA in immunity against *Brucella* and the influence of the expression of these splicing variants on the survival of this pathogen.

Given that we found no significant differences in the expression of other immunomodulatory lncRNAs like Malat1, Lethe, lincRNA-EPS, or Gas5 in BMM at 24 h post-infection, and considering that *B. abortus* modulates the macrophage response to evade its degradation and reach its replicative niche during the first 12 h post-infection (58, 62), we hypothesize that these immunomodulatory lncRNAs are differentially expressed by macrophages in early stages of the *Brucella* intracellular cycle. As we did not find any transcriptome RNA-seq data of primary macrophages after the first hours of *Brucella* infection, we used available RNA-seq data of RAW264.7 macrophages at 8h post-infection with *B. abortus* (37) to analyze the expression profile of lincRNA-Cox2, Lethe, lincRNA-EPS, Gas5 or Malat1, identifying changes in the abundance of the various transcripts associated with these lncRNAs. To assess whether this expression profile is specific to this stage or persists during infection, we performed the same analysis using data from RAW264.7 macrophages at 24 h post-infection with *B. abortus* (37). We identified 72 transcripts associated with the 5 lncRNAs studied, present in the bulk transcriptome data of RAW 264.7 cells at 8 h and 24 h post-infection (**Figure 2A**).

Of the 2 Lethe splicing variants detected, the only one to be differentially expressed was ENSMUST00000119415.2, which was overexpressed only at 8h post-infection, a period related to escape from phagolysosome degradation (62). The only lincRNA-EPS variant detected was ENSMUST00000247239.1 which decreased its expression only at 24h post-infection. Lethe and lincRNA-EPS are lncRNAs responsible for regulating inflammation. On the one hand, Lethe expression is promoted by NF-kB activation, and its function is to inhibit it, acting as a regulatory feedback loop. It has been previously reported that overexpression of Lethe in macrophages can inhibit the increase of ROS induced by high glucose conditions. On the other hand, lincRNA-EPS acts as a repressor of the expression of immune response genes in unstimulated bone marrow-derived macrophages (BMDM), mediating the interaction between promoters and topologically associating domain boundaries (23). It was recently reported that LincRNA-EPS-/- mice are less susceptible to lethal doses of Listeria monocytogenes and exhibit increased expression of proinflammatory cytokine genes, as well as increased expression of inducible nitric oxide synthase (iNos) and nitric oxide (NO) production. This suggests that Lethe and lincRNA-EPS may be involved in the survival of *Brucella* by influencing the inhibition of microbicidal mechanisms.

LincRNA-Cox2 is upregulated by the activation of NF-kB in response to inflammatory stimuli and participates in both the activation and repression of inflammatory genes. It is interesting to note that the expression of the ENSMUST00000241946.1 (a splice variant of lincRNA-Cox2) was the only one among the 72 variants analyzed to be overexpressed in RAW264.7 cells both at 8h and 24h post-infection. Although most of the lincRNA-Cox2 variants were overexpressed in RAW cells at 24h post-infection (**Figure 2B**), where we also found one of the variants overexpressed in BMM (**Figure 1B**), the overexpression of the ENSMUST00000241946.1 variant at 8h suggests that in early stages the inflammatory response is not completely inhibited by infection since NF-kB-dependent lncRNAs such as lincRNA-Cox2 (63) are being expressed.

Expression analysis revealed different profiles between 8 h and 24 h post-infection, also showing an increase in the diversity of differentially expressed splice variants at 24h post-infection, as observed with the expression of Gas5 (**Figure 2C**). Previous studies have demonstrated the involvement of Malat1 in the regulation of the alternative splicing process (33, 64). We observe that Malat1 exhibits a decrease in expression at 8 h post-infection, followed by an increase in the count of the same transcript at the 24 h post-infection, which could be related to the increase in the number of splicing variants observed. It is still unclear whether the various splicing variants of the same lncRNA are produced by the same cell or whether they are the result of heterogeneous infection-induced phenotypes as proposed by some authors (65) which could suggest an increase in phenotype diversity during the progression of the infection. Future studies using single-cell sequencing may resolve this question, and although knowledge about the functional role of lncRNAs and each of the variants is still scarce, evidence linking the dysregulation of alternative splicing of lncRNAs to the onset of diseases accumulates every year, positioning the analysis of lncRNA splicing variants as promising biomarkers for prognosis, diagnosis (66) and introducing a new and interesting aspect to help understand the complex machinery that regulates the immune response against *B. abortus*.

During the onset of infection, *B. abortus* modulates the response of invaded macrophages, impairing the activation of MyD88-dependent signaling pathways and reducing the inflammatory activity triggered by pathogen recognition. The immunomodulatory role of lincRNA-Cox2, Lethe, lincRNA-EPS, Gas5 and Malat1 in response to inflammatory stimuli has been previously reported in different inflammatory models, however, to date there are no reports linking them to the regulation of the immune response in response to *Brucella* infection. Our analysis of transcriptomic data from macrophages infected with *B. abortus* suggests that during different stages of infection, these lncRNAs exhibit a distinct expression pattern. To evaluate the influence of these lncRNAs in modulating the immune response during *Brucella* infection, we elaborated an interaction network between lnmunity-related genes and genes related to the lncRNAs evaluated here (**Figure 3**). The analysis shows that most of them are interactions showing a physical association of the protein-protein and protein-DNA type and many of them related to transcriptional regulation. Some interaction networks formed by genes such as Ifnb1 or cxcl2, stand out for showing a large number of associated interactions (**Supplementary Data 3**), suggesting that the modulation of the expression of both genes are affected by changes in the expression of these lncRNAs during *B. abortus* infection.

Differences in the expression profile of the five lncRNAs studied here are observed between 8h and 24h post-infection, and previous studies have shown that during the first 12h of infection *B. abortus* modulates the immune response to evade phagolysosome degradation and establish its replicative niche (4, 62). Hence, we used qPCR to evaluate the expression of lincRNA-Cox2, Lethe, lincRNA-EPS, Malat1, and Gas5 during the first 12 h of infection in RAW 264.7 cells. Specifically at 1 and 6 h, no significant changes were observed in the expression of any of the five evaluated lncRNAs (**Figure 4**). This suggests that they may not play a prominent role in the early stages of the immune response, at least in the context of *B. abortus* infection in this cell type. However, it is essential to recognize that the immune response is a highly dynamic process (67), and although these lncRNAs do not actively participate in the early stages, they could be crucial in subsequent phases. This hypothesis is supported by the significant increase in the expression of Lethe at 12 h post-infection (**Figure 4B**). This increase suggests that could be participating in the modulation of the inflammatory response in intermediate stages of infection, possibly acting as a negative feedback mechanism to avoid an excessive immune response that could be harmful to the host (19). Lethe overexpression occurs exclusively in response to S2308 infection and not in response to RB51 or HKBA infection, reinforcing the idea of their participation in the modulation of the immune response and their possible role in promoting an environment that facilitates the escape of the bacteria from degradation. Note that the overexpression of Lethe detected by qPCR at 12h post-infection is consistent with what we observed in our previous bioinformatic analysis (**Figure 2B**). On the other hand, both LPS stimulation or S2308 infection induced the expression of lincRNA-Cox2 at 12 h post-infection. However, in contrast to infection, the LPS-treated group induced lincRNA-Cox2 overexpression at 6 h post-stimulation (**Figure 4A**). This response correlates with the levels of cytokines evaluated, where while the infection does not induce changes in IL-6 production and only stimulates an increase in TNF-α at 12 h post-infection, the LPS-treated group induces an increase in the secretion of both cytokines from 6 h post-stimulation (**Figures 6A, B**). It was recently reported that lincRNA-Cox2 knockdown suppressed LPS-induced inflammation and M1 macrophage marker expression, and promoted M2 macrophage marker expression in primary peritoneal macrophages and RAW264.7 cells (68), suggesting that the expression of this lncRNA is modulated to favor the opening of a window in the activation of inflammation that allows *Brucella* to escape degradation.

Considering the lack of studies on the expression of lncRNAs during primary macrophage line infection and the changes observed in the expression of the five lncRNAs in RAW 264.7 cells after 12 h post-infection, we evaluated their expression in resident murine peritoneal macrophages after 12 h post-infection. Similar to what was observed in RAW264.7 cells, we found that lincRNA-cox2 was overexpressed in response to S2308 infection (**Figure 5A**), a bacterium that is mainly recognized by TLR2, TLR4 and TLR9 (24), with the expression of this lncRNA being independent of the viability of the bacterium. This phenomenon was also observed in response to RB51 strain infection and LPS stimulation. All of this is consistent with previous reports showing that lincRNA-Cox2 is overexpressed in a MyD88-dependent manner in response to the activation of various TLRs such as TLR2, TLR4, TLR7/8, or in response to infection with *Listeria monocytogenes* (63). Furthermore, this is suggestive of lincRNA-Cox2 being a key lncRNA in the regulation of the NF-kB-regulated inflammatory response and possibly actively participating during the response induced by *B. abortus*. On the other hand, although RB51 infection or HKBA stimulation downregulated the expression of Lethe and lincRNA-EPS, no significant changes were observed in these lncRNAs in peritoneal macrophages after infection with S2308 (**Figures 5B, C**). This suggests that even during this stage of infection, both lncRNAs could continue to exert regulatory roles in inflammation, favoring the intracellular viability of *Brucella*. Interestingly, in this model, Malat1 was downregulated in response to infection (**Figure 5E**), similar to what was observed in our bioinformatics analysis where Malat1 was downregulated in RAW 264.7 cells at 8h post-infection, a phenomenon that could be contributing to the delay in the induction of inflammation, considering that in other studies, Malat1 knockdown reduces the expression of proinflammatory cytokines such as IL-6 and TNF-α, also decreasing cell apoptosis (30, 31), partially explaining the response to inhibition of apoptosis by the virulent *B. abortus* 2308 strain (69). In addition, Gas5 was also downregulated (**Figure 5D**), an interesting result considering that Gas5 knockdown reduces inflammation and oxidative stress production in macrophages (70), key biological processes for the clearance of *Brucella* (71).

So far, previous studies that have analyzed the expression profile of lncRNAs in response to *Brucella abortus* infection have described how the alteration of the expression pattern of certain lncRNAs such as LNC_000428 and Gm28309, decrease the activation of inflammation favoring intracellular replication of *Brucella* in murine macrophages, suggesting protective functions of these lncRNAs during infection (36, 37). Our research contributes to this characterization by focusing on the analysis of the expression of five lncRNAs with reported immunomodulatory activity, revealing that the natural pattern of expression of these lncRNAs induced by infection is related to the stealth strategy used by *Brucella* during the onset of invasion to survive and replicate inside macrophages. The ability of *Brucella* to evade TLR4 activation at 6 hours and then activate TLR4, TLR2 and MyD88 at 24 hours post-infection, probably by action of its atypical LPS or by secretion of effectors through its T4SS (72, 73), could explain why the expression of some inflammation-promoting lncRNAs such as LincRNA-Cox2, increases only after 12-24 hours post infection, or why phenomena such as lack of decreased or increased expression of LincRNA-EPS and Lethe, whose expression plays a role of transcriptional brake of some proinflammatory cytokines and chemokines or repressor of NF-kB translocation to the nucleus, respectively, are observed only during infection of *B. abortus* 2308 and not in response to infection with the rough *B. abortus* RB51 strain or stimulation by HKBA.

Although our research introduces a new and interesting approach to the study of the modulation of the immune response by *Brucella*, these results should be complemented with more in-depth transcriptional studies incorporating larger sample sizes (N), as well as the use of primary lines or *in vivo* models to avoid the limitations of using cell lines such as RAW264.7, which do not express the ASC protein (74), widely used in the study of lncRNAs related to immunity, and thus strengthen the validity and generalization of the data, improving the robustness of the conclusions obtained in this initial study.

Considering the above, an interesting perspective to follow is the use of the expression of lncRNAs as markers for the diagnosis of Brucellosis. Although a couple of studies have recently explored this guideline (75, 76), there is a need to increase the evidence to support their use in the clinic. In this study we identified that lincRNA-Cox2 is significantly overexpressed during infection in three different macrophage types. One of its splicing variants (ENSMUST00000241946.1) is overexpressed in RAW 264.7 macrophages at both 8h and 24 h post-infection, suggesting that specific lincRNA-Cox2 splicing variants could be good diagnostic marker candidates.

Even though the results of these investigations are still exploratory, with the passage of time, the decrease in the cost associated with the execution of massive sequencing assays and the gradual incorporation of new technologies such as the characterization of expression profiles by Single Cell RNA-Seq together with the integration of Deep Learning tools (77), offer a promising platform for the development of a new generation of clinical markers based on the identification of transcriptomic expression patterns that allow more precise and personalized diagnoses to be obtained. On the other hand, it is imperative to increase the knowledge of lncRNAs both mechanistically and functionally, and to individualize the study of each of their splicing variants, which, as in the case FL and C2 splice variant of Gas5, could have divergent functions (78). In addition to the above, the identification of interaction networks between lncRNAs and other molecules during infection, approached from a spatiotemporal perspective, will allow progress in the development of gene therapies that help to equalize and direct the immune response more precisely and safely.

## Data availability statement

The original contributions presented in the study are included in the article/**Supplementary Material**, further inquiries can be directed to the corresponding author/s.

## Ethics statement

The animal study was approved by Ethics, Bioethics and biosafety committee. The study was conducted in accordance with the local legislation and institutional requirements.

## Author contributions

MF: Conceptualization, Investigation, Software, Writing – original draft. LG: Investigation, Writing – review & editing. RS: Investigation, Writing – review & editing. RM: Investigation, Writing – review & editing. RC-R: Investigation, Writing – review & editing. DM: Investigation, Writing – review & editing. ÍF: Investigation, Writing – review & editing. ÁO: Funding acquisition, Supervision, Writing – review & editing.
